# Outcomes for congenitally corrected transposition of great arteries using various surgical techniques: a systematic review and network meta-analysis

**DOI:** 10.1308/rcsann.2026.0019

**Published:** 2026-03-05

**Authors:** SU Hasan, SUR Usmani, SH Ahmed, A Pervez, MA Kamalia, AD Shah, MM Zubair

**Affiliations:** ^1^ Nottingham University Hospitals NHS Trust, UK; ^2^ Dow University of Health Sciences, Karachi, Pakistan; ^3^ Geisinger Health System, Danville, USA; ^4^ Medical College of Wisconsin, Milwaukee, USA; ^5^ University of Minnesota, Minneapolis, USA; ^6^ Children’s Mercy, Kansas City, USA

**Keywords:** Abnormality, Congenital, transposition of the great vessels, Congenitally corrected, transposition of the great arteries, Congenitally corrected, Cardiac surgery, Heart transplantation

## Abstract

**Introduction:**

Congenitally corrected transposition of the great arteries (ccTGA) is a rare disorder. Here, we evaluated the effectiveness of physiological, anatomical and Fontan repair techniques for ccTGA.

**Methods:**

A systematic database search in PubMed, Embase and Cochrane Central Register of Controlled Trials was conducted from inception to 5 June 2023. Random-effects network meta-analysis was performed, comparing mortality, postoperative arrhythmias, reintervention after definitive surgery and transplant-free survival in patients undergoing physiological, anatomical and Fontan repair.

**Findings:**

A total of 1,209 patients were included from nine studies. Fontan repair was associated with a higher rate of transplant-free survival (absolute risk [AR] 0.999 (±0.0101), 95% confidence interval [CI]) and reintervention after definitive surgery (AR 0.098 (±0.067), 95% CI), whereas the difference for mortality and postoperative arrhythmias did not reach statistical significance.

**Conclusions:**

Fontan repair is significantly better than anatomical or physiological repair in terms of transplant-free survival and also had a better rank probability for overall survival and reintervention after definitive surgery.

## Introduction

Congenitally corrected transposition of the great arteries (ccTGA) is a rare disorder; it is characterised by discordance between the atrioventricular and ventriculoarterial morphology.^1,2^ Although it allows for normal circulation, it is associated with several defects such as ventricular septal defect, pulmonary stenosis, tricuspid valve anomalies or pulmonary atresia (PA). A patient with ccTGA can present with a varying picture depending on the type and extent of the associated defect. Thus, the intervention can be subjective to clinical presentation of the patient. Current strategies include conservative management, physiological repair, anatomic repair, Fontan repair and primary heart transplant.^3^

‘Physiological repair’ aims to maintain the morphological right ventricle (RV) in the systemic position while repairing all the associated defects; however, the current literature suggests that long-term outcomes include progressive right ventricular dysfunction and tricuspid insufficiency.^4–6^ ‘Anatomic repair’ focuses on restoring the morphology of the heart by repairing the discordance and restoring the left ventricle as the systemic ventricle; the current literature provides both the merits and demerits of anatomic repair. Although some studies show excellent intermediate- and medium-term survival, others report several late complications such as RV to PA conduit stenosis, progressive aortic insufficiency and atrial switch pathway obstruction.^7–9^ The third option is a ‘single-ventricle Fontan pathway’ in the form of a total cavo-pulmonary connection.

There is currently no consensus on which surgical intervention is the best to treat patients with ccTGA. To our knowledge, the current study is the first of its type, with no prior network meta-analysis published on this topic. The purpose of the current study is to perform an extensive literature review of all the available studies/trials and perform a meta-analysis regarding predefined parameters such as mortality, postoperative arrhythmias, reintervention after definitive surgery and transplant-free survival, thus providing statistical evidence on which approach shows better results.

## Methods

### Selection criteria

This is a network meta-analysis based on previously published studies and therefore does not require ethical approval and patient consent. This article was written and completed according to PRISMA guidelines.

The inclusion criteria for selected articles were: (1) studies that included patients with ccTGA; (2) studies focusing on different corrective techniques for ccTGA, namely Fontan repair, anatomical repair and physiological repair; (3) studies that reported postoperative mortality, postoperative arrhythmias, reintervention after definitive treatment and transplant-free survival; and (4) randomised controlled trials (RCTs) and observational studies (including both retrospective and prospective studies).

Case reports, case series or articles that mentioned data in the form of an odds ratio (OR) or hazard ratio were excluded. Articles from which only single-arm data extraction was possible despite them mentioning two surgical techniques were excluded. Surgical techniques mentioned and compared in fewer than three articles were excluded. Articles in any other language besides English were also excluded.

### Data collection

Two independent investigators conducted the literature review following the predefined inclusion criteria. All disagreements were resolved by an unbiased third author. The following terms were combined using the Boolean operator (AND and OR): “ccTGA”, “Congenitally corrected transposition of the great arteries”, “Conventional”, “Physiological”, “Anatomic”, “Fontan”, “Heart transplant”. The literature review was conducted on PubMed, Embase and Cochrane Central Register of Controlled Trials from inception to 5 June 2023.

### Statistical analysis

To perform the network meta-analysis, we used a Bayesian random-effects model with a non-informative prior distribution and large variance (10,000), combining multiple nodes simultaneously, and creating evidence with direct and indirect comparisons.^10–12^ The data in the current study were in binary form, thus we used OR and absolute risk (AR) to calculate mean and standard deviation, along with creating an absolute plot, contrast plot and density plot with 95% confidence intervals (95% CI) using Markov Chain Monte Carlo simulation with 3 parallel chains of 200,000 simulation iterations. A node split model was utilised to assess inconsistencies using DIC, D.bar and pD; because of the small sample size, the model was specified as heterogeneous for random effects, and the null hypothesis was used for heterogeneity.^13–15^ Heterogeneity was calculated with Cochran’s Q test using the package ‘DescTools’, Higgins *I*^2^ test was computed using the Cochran’s Q value, and low heterogeneity was defined as *I*^2^ < 50%, *p* > 0.1. Owing to the small sample size, we assigned an inverse-Wishart before the variance–covariance matrix of random effects. Sensitivity analysis was performed by excluding one study at a time to find the source of heterogeneity where heterogeneity was significant.^16,17^ Risk difference (RD) was used to calculate the rank probability for all the parameters.^18^ We performed the analysis on RStudio version 1.4.1717 using the ‘pcnetmeta’ package, a programming language for statistical computing and graphics.

### Quality assessment

Quality assessment was undertaken by two independent investigators using the Newcastle–Ottawa Scale (NOS) to assess the quality of non-randomised studies. A single asterisk (*) represented one star awarded for meeting the specified criterion. The Comparability domain allows a maximum of two stars (**) for adequate control of confounding factors. The maximum possible score was 9 stars.

## Findings

### Study selection

**Figure 1:**
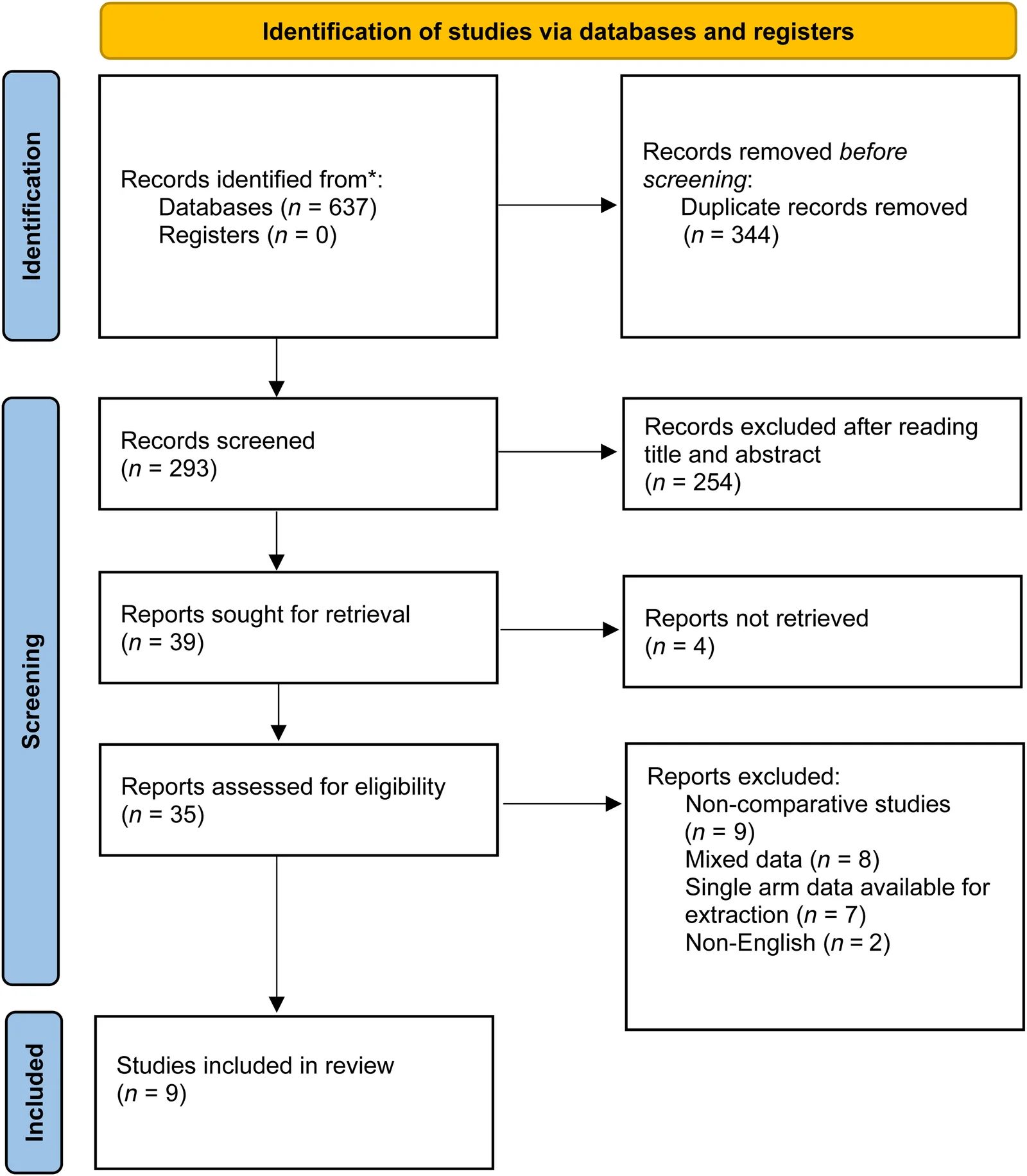
Identification, screening and inclusion of articles according to the PRISMA guidelines

**Figure 2:**
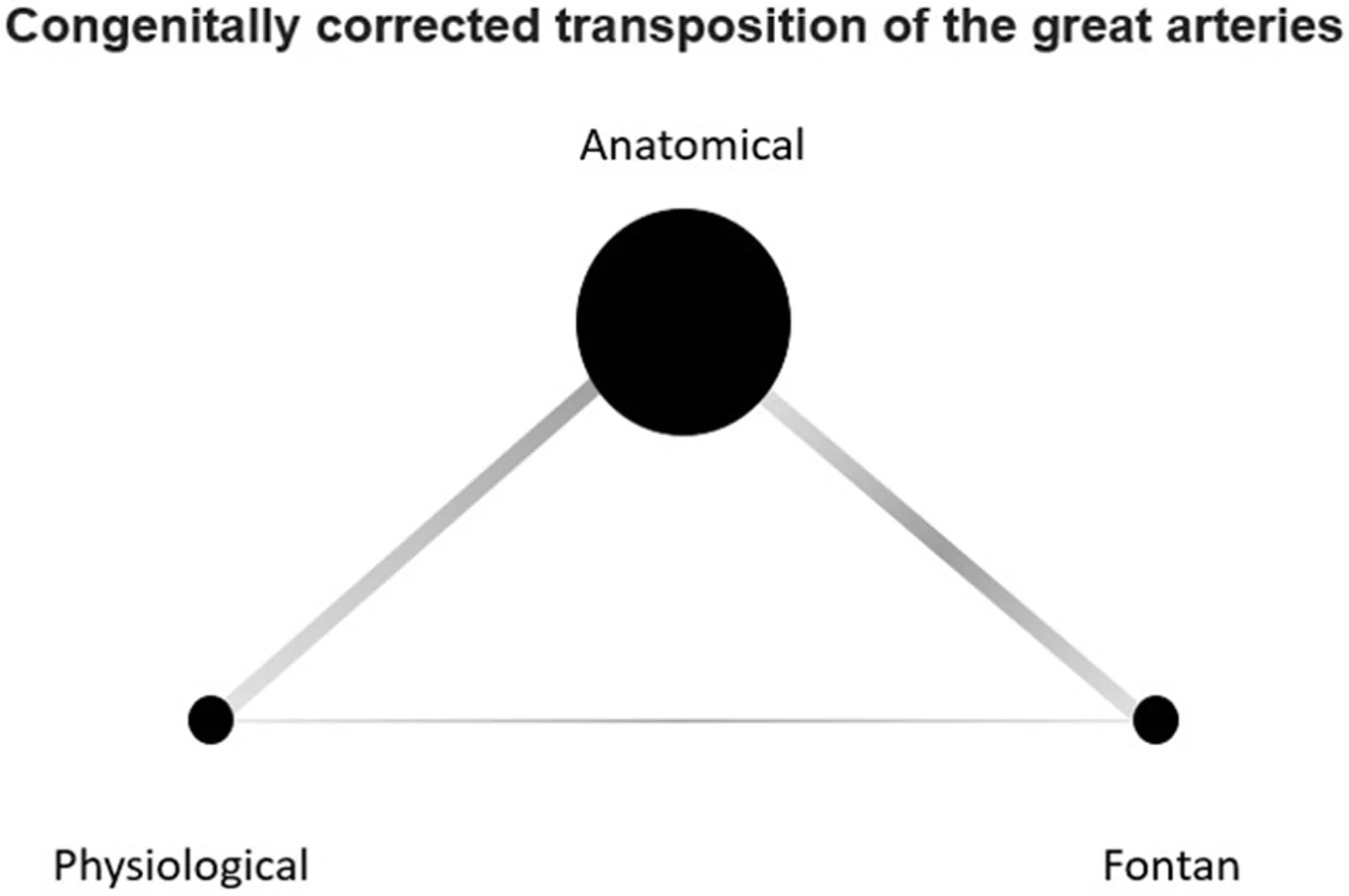
Network plot. The size of the circle denotes the number of studies and the thickness of the line between any two circles denotes the number of studies comparing the two interventions.

In total, 637 relevant studies were identified for screening after the literature review. After the exclusion of duplicates, 293 unique trials were engaged, of which 254 were excluded following stage 1 (title/abstract) screening. After stage 2 (full text) screening of the remaining 39 trials, 27 were omitted because they did not meet the inclusion criteria. Nine trials met our full inclusion criteria (Figure 1).^19–27^ A network plot was constructed to represent the studies (Figure 2).

### Study characteristics

All the included articles were comparative studies; eight trials used anatomic repair, five used physiological repair and five used Fontan repair for the surgical repair of ccTGA. The included 9 trials consisted of 19 arms comprising 1,209 patients, with the majority of the patient population belonging to the paediatric age group (Table 1).

**Table 1:** Demographics

Author, Year	Surgical treatment	Number of patients	Patient age (years)	Patient weight (kg)	Follow-up time (years)
Horer, 2008	Anatomic	6	3.5	NA	7
	Physiological	39	13.4	NA	8.2
Lim, 2009	Anatomic	44	NA	NA	5.9
	Physiological	123	NA	NA	4.6
Hsu, 2016	Anatomic	18	8.4	22.7	4.9
	Physiological	15	16.8	29.7	5.79
Leon, 2017	Anatomic	26	3	12.4	NA
	Physiological	45	6	14.7	NA
	Fontan	9	4	14.9	NA
Marathe, 2017	Anatomic	12	2.6	12.5	8.7
	Fontan	7	7.2	23.2	6.3
Sun, 2019	Anatomic	100	1.8	NA	7.3
	Fontan	29	5.4	NA	5.4
Ma, 2020	Anatomic	11	1.1	8.5	3.4
	Fontan	13	2.1	14.6	4.3
He, 2021	Anatomic	41	2.5	12.5	5.1
	Physiological	38	2.1	16.4	5.1
Chew, 2022	Anatomic	400	NA	NA	NA
	Fontan	233	NA	NA	NA

NA = not available

### Efficacy outcomes

#### Mortality

**Figure 3:**
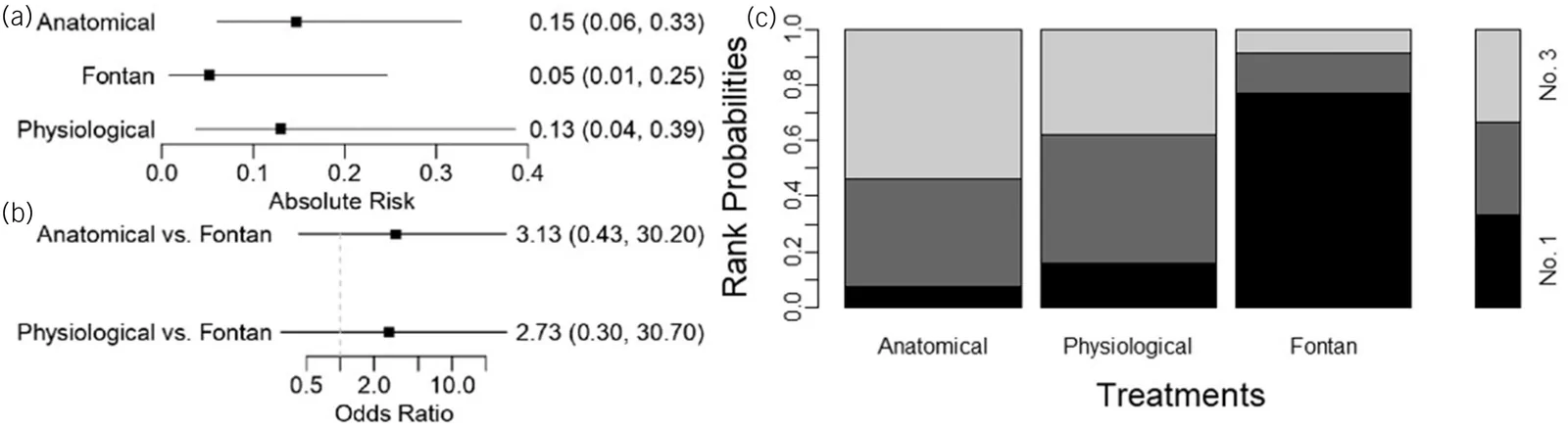
(a) Absolute risk. Absolute plot comparing all the treatments for the number of mortalities. Although there is no significant difference between the treatments, Fontan shows the lowest absolute risk of 0.05 (0.01, 0.25) giving it the highest probability for the lowest mortality rate. (b) Contrast plot comparing all the treatments with Fontan for the number of mortalities using the odds ratio shows no significant difference between Fontan and other treatments. (c) Rank probability, calculated using risk difference, computed as a bar chart shows a probability of 77% for Fontan to be the best treatment for the lowest number of mortalities.

Seven studies consisting of 14 arms reported this outcome, and all the results were reported with 95% CI.^19,21,23–27^ The mean was calculated using AR, with Fontan showing the lowest mean value of 0.071 (±0.063), followed by physiological repair with a mean value of 0.151 (±0.090) (Figure 3a). OR was used to construct a contrast plot comparing all the treatments with Fontan; the results showed no significant difference among the treatments (Figure 3b). RD was used to calculate the rank probability for all the treatments. Fontan showed the most promising result, with a 77% probability of being the best treatment, followed by physiological repair with a probability of 15.7% and anatomic repair in third with a probability of 7.25% (Figure 3c). Significant heterogeneity was observed (*I*^2^ = 89%, *p* = 0.0004). Sensitivity analysis was performed to determine the source of heterogeneity but this could not be determined.

#### Postoperative arrhythmia

**Figure 4:**
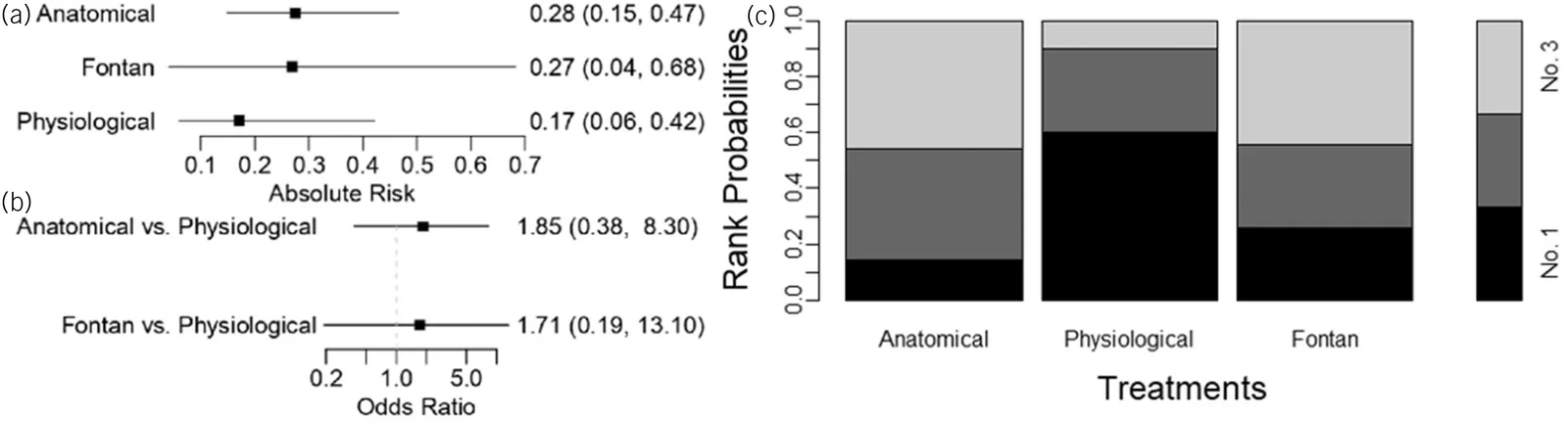
(a) Absolute risk. Absolute plot comparing all the treatments for postoperative arrhythmias. Although there is no significant difference between the treatments, physiological repair shows the lowest absolute risk of 0.17 (0.06, 0.42) giving it the highest probability for the lowest postoperative arrhythmia rate. (b) Contrast plot comparing all the treatments with physiological repair for the number of postoperative arrhythmias using the odds ratio shows no significant difference between physiological repair and other treatments. (c) Rank probability, calculated using risk difference, computed as a bar chart shows a probability of 60% for physiological repair to be the best treatment for the lowest number of postoperative arrhythmias.

Five studies consisting of 11 arms reported this outcome, and all the results were reported with 95% CI.^20–23,27^ The mean was calculated using AR, with physiological repair showing the lowest mean value of 0.189 (±0.092), followed by anatomical repair with a mean value of 0.283 (±0.081) (Figure 4a). OR was used to construct a contrast plot comparing all the treatments with physiological repair; the results showed no significant difference among the treatments (Figure 4b). RD was used to calculate the rank probability for all the treatments. Physiological repair showed the most promising result with a 60% probability of being the best treatment (Figure 4c). Significant heterogeneity was not observed (*I*^2^ = 19%, *p* = 0.29).

#### Reintervention after definitive surgery

**Figure 5:**
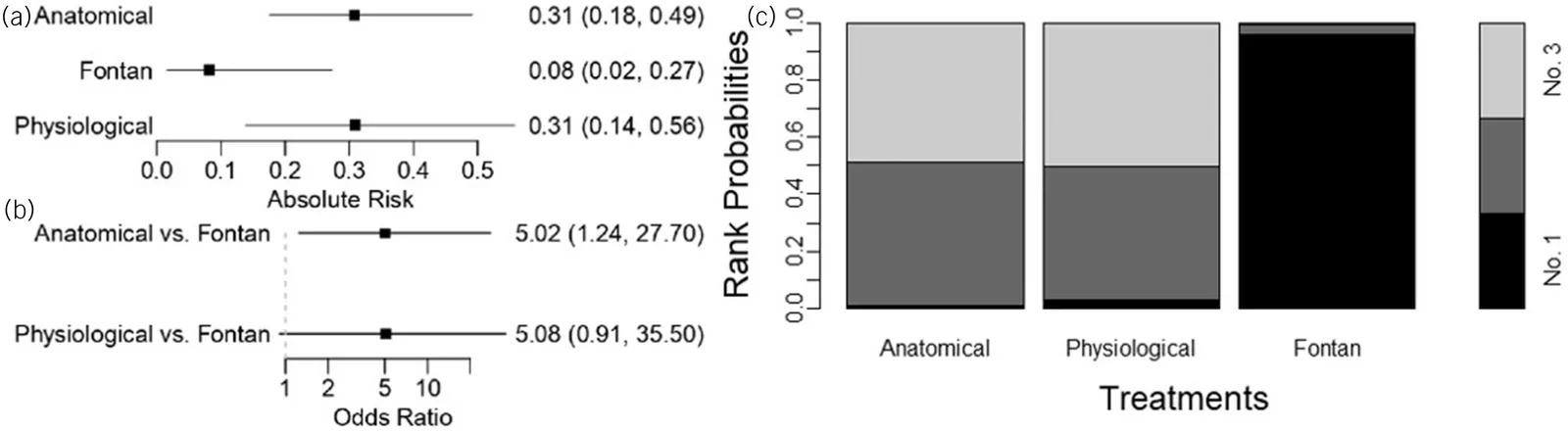
(a) Absolute risk. Absolute plot comparing all the treatments for the number of reinterventions after definitive surgery. Although there is no significant difference between the treatments, Fontan shows the lowest absolute risk of 0.08 (0.02, 0.27) giving it the highest probability for the lowest number of reinterventions after definitive surgery. (b) Contrast plot comparing all the treatments with Fontan for reintervention after definitive surgery using the odds ratio shows a significant difference between Fontan and anatomical repair while no significant difference between Fontan and physiological repair. (c) Rank probability, calculated using risk difference, computed as a bar chart shows a probability of 96.2% for Fontan to be the best treatment for the lowest probability of reintervention after definitive surgery.

Eight studies consisting of 17 arms reported this outcome, and all the results were reported with 95% CI.^19–26^ The mean was calculated using AR, with Fontan showing the lowest mean value of 0.098 (±0.067) (Figure 5a). OR was used to construct a contrast plot comparing all the treatments with Fontan; the results showed a significant difference between Fontan and anatomical repair but a non-significant difference between Fontan and physiological repair (Figure 5b). RD was used to calculate the rank probability for all the treatments, with Fontan showing the most promising result with a 96.2% probability of being the best treatment, followed by physiological repair with a probability of 2.95% and anatomic repair in third with a probability of 0.88% (Figure 5c). Significant heterogeneity was not observed (*I*^2^ = 7%, *p* = 0.31).

#### Transplant-free survival

**Figure 6:**
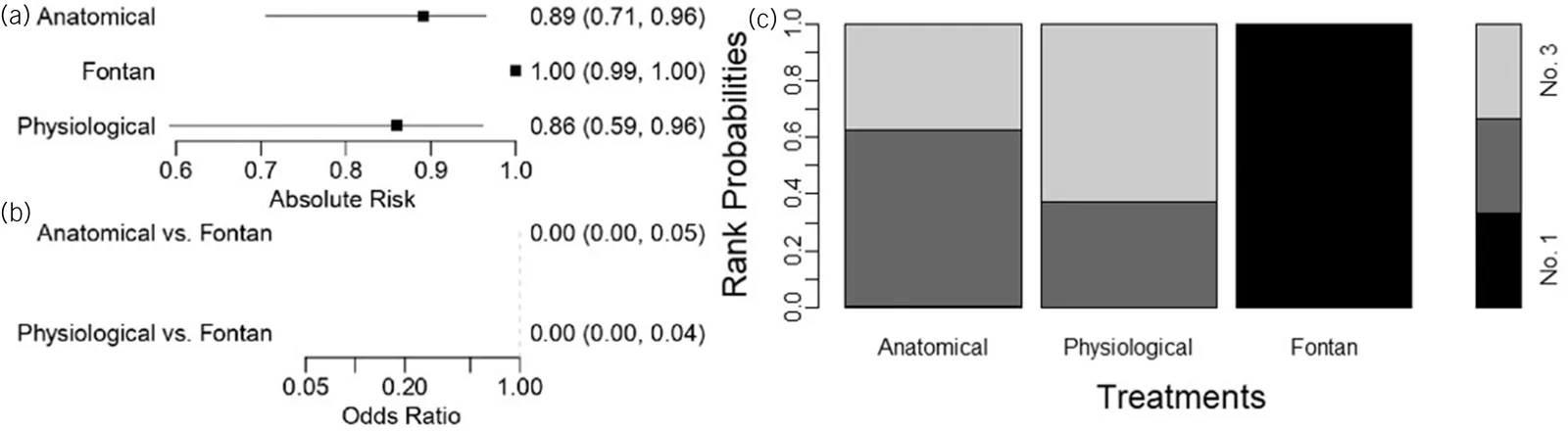
(a) Absolute risk. Absolute plot comparing all the treatments for transplant-free survival. There is a significant difference between Fontan and the other treatments because Fontan shows the highest absolute risk of 1.00 (0.99, 1.00) giving it the highest probability for transplant-free survival. (b) Contrast plot comparing all the treatments with Fontan for transplant-free survival using the odds ratio shows a significant difference between Fontan and other treatments. (c), Rank probability, calculated using risk difference, computed as a bar chart shows a probability of 99.7% for Fontan to be the best treatment for transplant-free survival.

Five studies consisting of 11 arms reported this outcome, and all the results were reported with 95% CI.^19,21,22,24,25^ The mean was calculated using AR, with Fontan showing the highest mean value of 0.999 (±0.010) (Figure 6a). OR was used to construct a contrast plot comparing all the treatments with Fontan. The results showed a significant difference between Fontan and the other two treatments (Figure 6b). RD was used to calculate the rank probability for all the treatments, with Fontan showing the most promising result with a 99.7% probability of being the best treatment (Figure 6c). Significant heterogeneity was not observed (*I*^2^ = 24%, *p* = 0.27).

### Quality assessment

Quality assessment was done using the NOS tool; according to the quality assessment of NOS all the studies were given a high score (7–9). The results are represented in Table 2.

**Table 2:** Newcastle–Ottawa scale for quality assessment.

Items	Selection				Comparability	Exposure			Total scores
	Adequacy of case definition	Representativeness of the cases	Selection of controls	Definition of controls		Ascertainment of exposure	Same method of ascertainment	Non-response rate	
Horer, 2008	*	–	*	*	*	*	*	–	7
Lim, 2009	*	*	–	–	*	*	*	*	7
Hsu, 2016	*	*	*	*	*	*	*	*	8
Leon, 2017	*	–	*	*	*	*	*	–	7
Marathe, 2017	*	–	*	*	**	–	*	*	7
Sun, 2019	*	*	*	*	*	*	*	–	8
Ma, 2020	*	*	*	*	**	*	*	–	8
He, 2021	*	*	*	*	*	–	*	*	8
Chew, 2022	*	*	*	*	**	*	*	*	9

* One star. ** Two stars (for comparability domain only).

## Discussion

To the best of our knowledge, this is the first meta-analysis discussing different surgical approaches for ccTGA, and although the appropriate management for patients presenting with ccTGA is a question of ongoing discussion, our study shows that Fontan repair is significantly better than physiological and anatomical repair with respect to transplant-free survival. Similarly, Fontan repair showed better trends for other outcomes as well.

There is one major variable that fabricates the heterogeneity in the clinical presentation of the patient and that is the variability present in the wide spectrum of lesions.^28^ Clinical presentation can vary from asymptomatic to impending heart failure depending on the type of cardiac lesion present, which is typically present in about 80% of patients with ccTGA.^29,^^30^ The only consistent feature present in all the patients is the discordance between atrioventricular and ventriculoarterial morphology. The different approaches that the current study discussed included ‘physiological repair’, ‘anatomic repair’ and ‘Fontan’. All the approaches have their merits and demerits as already mentioned in the introduction.

All the current surgical techniques used for ccTGA have some limitations; to assess those limitations we focused on the four most commonly reported parameters, which were mortality, postoperative arrhythmias, reintervention after definitive surgery and transplant-free survival. Although several other parameters can be used to assess the outcome of the surgical techniques, we used only the four most commonly reported outcomes.

The long-term survival rate reported by Hsu *et al* was 80% for physiological repair, 53% for anatomic repair and 100% for Fontan single ventricular palliation, compared with Horer *et al* who reported no significant difference between the treatments.^19,^^21^ Our study provides evidence that although there is no significant difference in rate of mortality among the treatments, the Fontan group had a better rank probability than the other two surgical options, as previously reported by Hsu *et al*.^21^ In the current study, Fontan repair reported the lowest mean AR of 0.151 (±0.090), thus giving the best chance of survival for the patient. Postoperative arrhythmia is one of the more common complications reported. Our analysis suggests that physiological repair is the safest choice to avoid postoperative arrhythmias, with a mean AR of 0.189 (±0.092), although there is no significant difference among the treatments.

The rate of reintervention is directly related to failure of the definitive surgery as well as the complication followed by reintervention. Previously published literature is suggestive of a high rate of intervention after physiological repair and a comparatively lower rate of reintervention after anatomic repair, presumably due to the incidence of complications.^5,30–33^ Horer *et al* suggested a very low rate of reoperation for Fontan repair, and similar results were reported by Hsu *et al*.^19,^^21^ Our study suggests that Fontan repair is significantly better than anatomical repair in terms of reintervention, with a mean AR of 0.098 (±0.067) and rank probability as high as 96.2%, making it the surgical procedure least likely to involve reintervention.

De Leon *et al* suggested that transplant-free survival is similar for all three types of surgical repair, and similar results were seen in the study presented by Sun *et al*.^22,^^24^ Although Fontan repair is considered to have excellent medium-term survival, it can result in long-term complications because of progressive ventricular dysfunction.^34,35^ Alomair *et al* suggested similar transplant-free survival for both anatomical and physiological repair.^36^ Our analysis provides evidence that Fontan repair with a mean AR of 0.999 (±0.010) is significantly better than both anatomical and physiological repair for transplant-free survival.

Our study has several strengths. The current article included only comparative studies, thus allowing us direct and indirect comparison between the parameters. To our knowledge, this is the first network meta-analysis comparing the three types of surgical procedures for ccTGA and providing statistics-based evidence regarding each parameter. Analysis of the data gave significant results and not just trends and ranks, thus providing surgeons with a conclusive result concerning which surgery should be preferred. Strict adherence to the inclusion and exclusion criteria meant that only relevant studies were included.

### Study limitations

Our study had some restrictions; we included only retrospective studies because ccTGA is a rare disorder and there is not enough literature in the form of prospective studies and RCTs. The sample size of the current study was small; ccTGA can present as a spectrum of clinical presentations, but this was not accounted for in our study. Anatomical repair can be of several types depending on the morphological abnormality, but the current study pooled all the data for anatomical repair. Surgical preference depends on the types of lesions associated with ccTGA and surgery can vary depending on the type of lesion present, but this was not accounted for in the pooled data.

## Conclusions

In conclusion, Fontan repair is significantly better than anatomical or physiological repair in terms of transplant-free survival. Fontan repair also had a better rank probability than the other two surgical options for overall survival and reintervention after definitive surgery. Surgeons should prefer Fontan repair wherever possible as the treatment of choice for ccTGA. For future comparative studies, it is important to adhere to a uniform pattern so that more studies can be included in the meta-analyses.

## Competing interests

The author/s declare no competing interests.

## Funding

The author/s received no financial support for the research, authorship and/or publication of this article.

## Ethics approval and consent to participate

Not applicable.

## Author contributions

**SU Hasan**: Conceptualization, Data curation, Formal analysis, Methodology, Project administration, Validation, Visualization, Writing – original draft, Writing – review & editing. **SUR Usmani**: Conceptualization, Data curation, Methodology, Project administration, Validation, Visualization, Writing – original draft, Writing – review & editing. **S Ahmed**: Data curation, Methodology, Validation, Writing – original draft, Writing – review & editing. **A Pervez**: Data curation, Validation, Writing – original draft, Writing – review & editing. **MA Kamalia**: Conceptualization, Methodology, Project administration, Supervision, Writing – review & editing. **AD Shah**: Data curation, Validation, Writing – original draft, Writing – review & editing. **MM Zubair**: Conceptualization, Methodology, Project administration, Supervision, Validation, Writing – review & editing.

## Artificial Intelligence

The author/s declare that no AI was used to conduct the study or prepare the manuscript.
